# Acute Ultra Low Dose THC Attenuates Anxiety-Like Behavior in a Mouse Model for Major-Depressive Disorder

**DOI:** 10.1007/s12031-026-02597-8

**Published:** 2026-10-12

**Authors:** Meitar Grad, Hadas Volkov, Noam Shomron

**Affiliations:** 1 Gray Faculty of Medical and Health Sciences, Tel Aviv University, Tel-Aviv, Israel; 2 Edmond J. Safra Center for Bioinformatics, Tel Aviv University, Tel-Aviv, Israel; 3 The Sagol School of Neuroscience, Tel Aviv University, Tel-Aviv, Israel

## Abstract

Major depressive disorder (MDD) is a prevalent psychiatric disorder, and is a leading cause of disability worldwide. Current treatments for MDD entail many side effects, and include a lag until the onset of therapeutic effects. The current hypotheses as for the etiology of MDD revolve around the monoamine hypothesis and the plasticity and neurodegeneration hypothesis. According to the latter, employing a neuroprotective treatment may help ameliorate depressive symptoms. Δ^9^-Tetrahydrocannabinol (THC) is the primary psychoactive component of the *cannabis sativa* plant. THC interacts with the endocannabinoid system, which was shown to be involved in the regulation of emotional states (e.g. anxiety) and stress through modulation of synaptic plasticity. Here, we show the potential of acute, ultra-low dose (0.002 mg/kg) THC in ameliorating anxious behavior in a chronic mild stress mouse model for MDD. We utilize next generation sequencing techniques to show the treatment’s effect on neuronal differentiation and synaptic plasticity-related mRNAs, and specify *Elmo domain containing 1* (*Elmod1*) as a molecular mediator of the observed anxiolytic effects. Moreover, we show the involvement of glucocorticoids-related mRNAs in the observed anxiolytic effect of acute, ultra-low dose THC.

## Introduction

Major depressive disorder (MDD) is a prevalent psychiatric disorder, and is a leading cause of disability worldwide (World Health Organization, 2025). MDD is characterized by low mood, anhedonia, disturbed sleep cycles and appetite, increased anxiety and impairments in cognition, among other symptoms, which untreated may lead to suicide (World Health Organization, 2025). The primary therapeutic approaches for MDD (e.g. selective serotonin reuptake inhibitors (SSRI’s)) are not always effective in reducing the depressive symptoms, and oftentimes include adverse side effects (Cartwright et al. 2016; Zhdanava et al. 2021). The etiology of MDD involves the combination of genetic predisposition, psychological endurance and environmental factors, such as stress (Page et al. 2024). While it had been largely asserted that instability of monoamines in the brain cause the symptoms of MDD (Jauhar et al. 2023), the current accepted dogma is that aberrations in neurogenesis and neuroplasticity mechanisms underly the pathophysiology of MDD (Page et al. 2024).

Chronic stress is a major contributor and catalyst for MDD (Boku et al. 2018; Cordner et al. 2021). Stress activates the hypothalamic-pituitary-adrenal (HPA) axis, which lead to a cascade of hormones that result in elevated glucocorticoid (GCs; i.e. cortisol) levels in the blood and cerebrospinal fluid (Boku et al. 2018; Turner 2024). When the GCs reach the hippocampus (HIP), they bind to GC receptors (GC-Rs) which lead to reduction of GC levels back to normal (Boku et al. 2018). Hence, the HIP is a critical player in the negative feedback of the HPA-axis (Boku et al. 2018; Cole et al. 2022). Previous findings have shown that in MDD, there is a substantial decrease in the volume of the HIP and a failure in the negative feedback loop of the HPA axis (de Kloet et al. 2005; Videbech and Ravnkilde 2004). This neurodegeneration is also accompanied by neuroinflammation in MDD patients (Tripathi et al. 2021; Wu and Zhang 2023). Importantly, chronic stress in rodents was also shown to induce neuroinflammation through the complement component 3-dependent pathway (Tripathi et al. 2021) and to be accompanied by disruptions to tryptophan metabolism, increases in pro-inflammatory cytokines and decreases in anti-inflammatory cytokines (Luo et al. 2025). Expanding on this, chronic stress in mice was shown to induce microglial activation and reduce neurogenesis in the dentate gyrus of the HIP (Du Preez et al. 2021). At the structural level, cytoskeletal dynamics play a critical role in neuronal structure, synaptic plasticity and stress responsiveness, all of which are disrupted in MDD (Wong et al. 2013). Importantly, it was shown that enhancement of neurogenesis in the brains of mouse models of MDD has ameliorated depression-like behaviors in those mice (Li et al. 2022; Planchez et al. 2021; Schloesser et al. 2015). According to this hypothesis, employing neuroprotective and anti-inflammatory agents may assist in treating MDD.

The chemical Δ^9^-Tetrahydrocannabinol (THC) is the primary psychoactive component of the *cannabis sativa* (marijuana) plant (Burggren et al. 2019; Kogan and Mechoulam 2007). THC interacts with the endocannabinoid system (Botsford et al. 2020; Kogan and Mechoulam 2007), which was shown to be involved in the regulation of emotional states (e.g. anxiety), stress and cognition through modulation of synaptic transmission and plasticity (Andrade et al. 2019; Botsford et al. 2020; Cooray et al. 2020; McEwen et al. 2015). THC primarily binds to cannabinoid 1 receptors (CB1-Rs) (Calabrese and Rubio-Casillas 2018; Fishbein-Kaminietsky et al. 2014), that are abundant in the central nervous system (CNS), with a significantly high density in the HIP (Calabrese and Rubio-Casillas 2018; Hungund et al. 2004) and cerebral cortex (Andrade et al. 2019; Hungund et al. 2004). Interestingly, CB1-R-deficient mice show depressive-like behaviors (Steiner et al. 2008), changes in depression-related genes (Aso et al. 2011), dysregulation of the HPA-axis (Steiner et al. 2008) and decreased hippocampal neurogenesis (Calabrese and Rubio-Casillas 2018; Jin et al. 2004). Thus, it was suggested that enhancement of CB1-R activity by THC may ameliorate these depressive-like behavioral and physiological changes (Calabrese and Rubio-Casillas 2018; Sarne 2019). Indeed, several studies have shown that THC exerts anxiolytic effects in rodents when administered via injection (*i.p.* or *sc.*) at relatively low doses (0.25-5 mg/kg THC) in conjunction with the stressor and/or immediately prior to behavioral testing (Bluett et al. 2017; Harte-Hargrove and Dow-Edwards 2012; Mayer et al. 2014; Uttl et al. 2018). Further studies indicated that treatment with low-dose THC ameliorated the stress-induced impairments in synaptic plasticity (Abush and Akirav 2013) and promoted HIP neurogenesis (Jiang et al. 2005) in the brains of treated rodents. Nevertheless, the anxiolytic effect was reversed when THC was injected (*i.p*.) at higher doses (10–15 mg/kg) (Harte-Hargrove and Dow-Edwards 2012; Kasten et al. 2017, 2019).

Previous research establishes that the functional outcome of acute, ultra-low dose injection of THC (0.001-0.002 mg/kg; 3–4 orders of magnitude lower than the conventional doses) is strictly dependent on the state of the brain. Rather than exerting a uniform effect, the acute, ultra-low dose of THC acts as a state-dependent modulator. In models of varied brain traumas, this specific dosage has been shown to protect against both underlying pathophysiology and subsequent cognitive decline. For example, acute administration of ultra-low dose THC 3 days after injection of the seizure-inducing agent pentylenetetrazole (PTZ) resulted in amelioration of the seizure-promoted cognitive deficits as observed in the dry maze and object recognition tests (Assaf et al. 2011). Similarly, administration of acute, ultra-low dose THC 7 days after the induction of neuroinflammation by lipopolysaccharide (LPS) injection protected against cognitive damage, as measured by the object recognition test (Fishbein-Kaminietsky et al. 2014). In addition, cognitive impairments induced by 3,4-methylenedioxymethamphetamine (MDMA) neurotoxicity were reversed when ultra-low dose THC was injected 1 day after MDMA treatment, as measured by the object recognition test (Fishbein et al. 2012). In line with these findings, acute, ultra-low dose THC protected against cognitive impairments resulted from carbon monoxide-induced hypoxia as seen in the dry maze (Fishbein et al. 2012). In all of these studies, acute, ultra-low dose THC showed therapeutic potential even when administered after the insult, and weeks before behavioral testing. This therapeutic potential extends to chronic neurodegenerative environments, such as aging. In old (24-months-old) female mice characterized by age-related cognitive decline, acute, ultra-low dose THC reversed spatial and object recognition deficits (Sarne et al. 2018), and was correlated with alterations in glucocorticoid-related genes expression (Shapira et al. 2023). Conversely, the critical nature of brain health context is highlighted by the finding that the same treatment in healthy, young (2-months-old) mice resulted in impaired cognitive performance (Tselnicker et al. 2007; Senn et al. 2008; Amal et al. 2010). Thus, the context of the brain’s health is critical for the effects of acute, ultra-low dose THC treatment; under healthy conditions it will have deleterious effects, while under brain trauma it will have beneficial effects (Sarne 2019).

Here, we show that acute, ultra-low dose THC was able to reduce anxiety in a chronic mild stress (CMS) model for MDD. We show that the treatment caused alterations in development-related transcripts in healthy, young mice hippocampi, and changes in neuronal differentiation and synaptic signaling pathways in CMS mice. Moreover, we suggest the involvement of the cytoskeleton-related gene *Elmo domain containing 1* (*Elmod1*), and glucocorticoids-pathway related genes *Serum and glucocorticoid kinase 1* (*Sgk1*), *Glycogen synthase kinase-3β* (*Gsk3β*) and *Nuclear receptor subfamily 3 group c member 1* (*Nr3c1*) in mediating the observed anxiolytic effects of acute, ultra-low dose THC.

## Methods

All experimental protocols were approved to be compatible with the standards for care and use of laboratory animals by the Tel-Aviv University Institutional Animal Care and Use Committee (approval number 01-21-074). All efforts were made to minimize animal suffering and the number of animals used.

### Animals

**Fig. 1:**
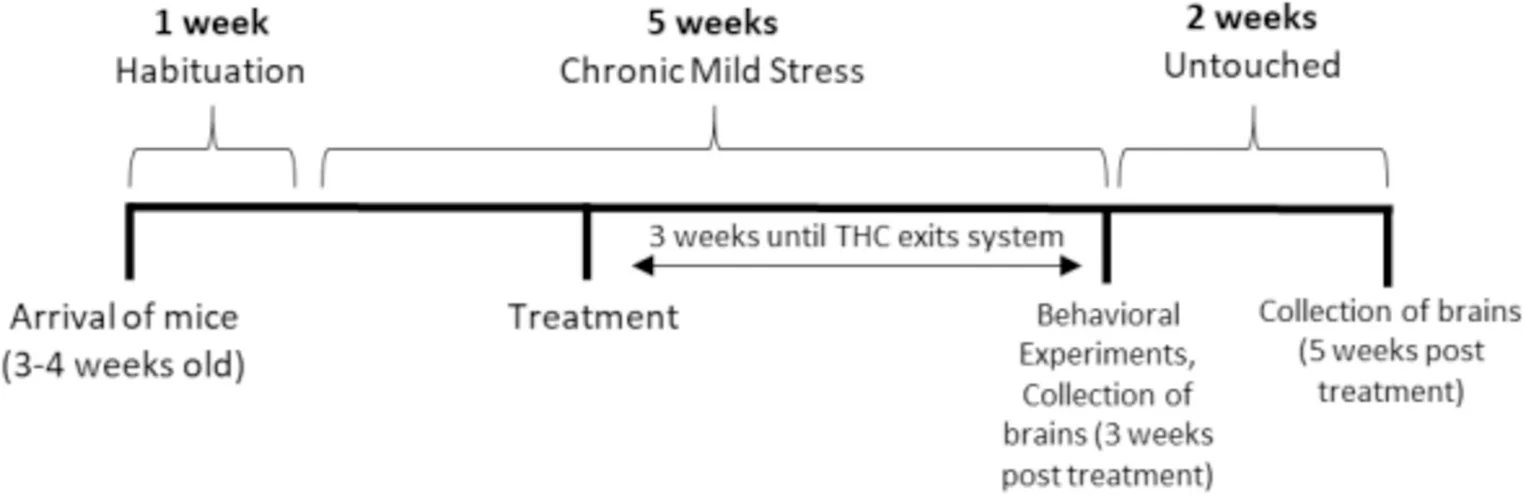
Timeline of experiments

Four-weeks-old male outbred ICR mice were purchased from the supplier (Harlan Laboratories Israel Ltd., Israel) and kept in the Tel-Aviv University Conventional Animal Facility at 23 °C, under a 12-hour light/dark cycle (7am lights on, 7pm lights off). This age of mice was chosen because previous research showed the deteriorating effect of acute, ultra-low dose THC on cognition at 2-months-old, which is the age the mice will be at testing, after the CMS protocol (Senn et al. 2008). Also, performing the CMS protocol in mice this age was shown to reduce neurogenesis in mice brains (Hill et al. 2012). Mice were housed in groups of 5 littermates per cage (unless under CMS protocol), with ad libitum access to food and water. After arrival to the animal facility, mice were marked according to their allocated number (Chen et al. 2016) (using a microtattoo system and ink; Fine Science Tools (FST), Germany) and allowed 1 week of habituation to their new surroundings before the beginning of the CMS protocol and treatments. For each cohort, half of the mice were chosen to undergo the CMS protocol, while the rest remained untouched (excluding ordinary cleaning and care) in their home-cage for the same duration. At the start of the third CMS week, mice were treated *i.p* with 0.002 mg/kg THC or vehicle, as described below. 3- or 5-weeks post treatment, once THC was eliminated from the mice’s systems, they were taken for behavioral testing or sacrificed for molecular analysis of their brains. Mice were 9–12 weeks old at testing (Fig. 1).

### Chronic Mild Stress (CMS) Protocol

The CMS protocol was implemented in accordance with previously reported methods (Hill et al. 2012; Willner 2017). Mice underwent a 5-week CMS regimen, in which they were housed alone in a cage for constant social isolation. Food and water were provided *ad-libitum*. During the CMS protocol, mice experienced 1–2 unpredicted transient stressors daily for 5 consecutive days out of each week. For the duration of the transient stressors, the mice stayed in their home cage but in a designated room, after which they were returned to their home room. The five transient stressors were as follows:

- *Forced bath* Mice were individually placed for 6 min in a container filled with 1 cm of water (pre-warmed to 37 °C), after which they were returned to their home cage and kept under a warm light for 6 min to dry.
- *Wet bedding* Clean water, warmed to 37 °C, was poured into each cage until bedding was thoroughly dampened without causing a pooling of water. The animals then remained in the damp cage for 2 h.
- *Cage tilt* Cages were tilted to an angle of 45 degrees for 2 h.
- *Cage switch* Mice were transferred to an empty cage of another mouse for 24 h, after which they were placed in a fresh cage.
- *Rat smell* Used bedding from rats’ cages were dispersed in mice’s home cages, and left there for 2 h, after which mice were transferred to a new cage.

### Treatments

Treatments were conducted in accordance with previously published literature (Sarne et al. 2018). Specifically, Mice randomized and were injected *i.p* with a solution of Saline (Baxter at TEVA, Israel) : Cremophor EL (Millipore, Merck, USA) : THC (T2386 at Sigma-Aldrich, USA) in a 18:1:1 ratio. THC was administered in a dose of 0.002 mg/kg. In vehicle-treated mice, THC was replaced with 100% Ethanol (Biolab, Israel), added to the saline and Cremophor solution. Injection volume was 0.1 ml/10 g body weight.

### Behavioral Assays

All behavioral assays were performed in a controlled environment at the Myers Neuro-Behavioral Core Facility in the Faculty of Medicine’s Animal Facility at Tel Aviv University. 30 to 60 min before each behavioral assay, mice were transferred in their home cages from their homeroom in the Animal Facility to a designated room in the Myers Neuro-Behavioral Core Facility for habituation. At the end of each behavioral testing day, the mice were returned to their homeroom, still in their familiar home cages. All behavioral assays were conducted in a separate, quiet room under dimmed lights during the mice’s light phase. Behavioral apparatuses were thoroughly cleaned with Virusolve+ (Amity International, UK) before and after testing of each mouse, to remove excrements and remaining odor. Mice behavior was recorded using the Basler GenIcam (Basler acA1300-60; Basler AG, Germany), with a frame rate of 25 frames per second and a resolution of 1280*960 pixels. The EthoVision XT 17 or EthoVision XT 13 softwares (Noldus Information Technology, The Netherlands) were used to process and analyze the recordings. Each mouse underwent 2 behavioral tests, with at least 24-hour rest between tests. The entirety of the behavioral testing lasted 1 week, at the end of which the mice were sacrificed.

#### Elevated Plus Maze

The elevated plus maze is used to assess mice’s anxiety, utilizing their innate aversion from open high spaces when contrasted with their inclination towards exploring novel surroundings. The elevated plus maze consists of a plus-shaped apparatus, with two opposing open arms and two closed arms (enclosed within 12 cm-tall walls). The maze is raised 1 m from ground level. Each mouse is put alone in the center of the maze and given 7 min to explore it freely, while being recorded. The duration (in seconds) and frequency of visits to open/closed arms and to edges of open arms were analyzed.

#### Open Field Test

Mice were introduced into the arena (always starting at the same corner), and allowed 15 min of free exploration. Their behavior was recorded, and the frequency and cumulative duration (in seconds) of entries to the center of the arena (noted as the central 25cm^2^ square-shaped area) were analyzed as a measure for anxiety levels. The Arena was composed of four acrylic sheet white walls measuring at 30 cm high and 50 cm wide, with 50cm^2^ black acrylic sheet for the floor.

### RNA

#### RNA Extraction

Tissue slices or cells were thoroughly homogenized in 1 ml TRIzol reagent (Invitrogen, Thermo-Fisher Scientific, USA) using the TissueLyser II homogenizer (Qiagen, Germany) or repeated pipettation, respectively. After incubation at room temperature for 5 min, 200 µL of chloroform (Bio-Lab Ltd., Israel) was added to each sample, and tubes were shaken manually for 15s. Following another incubation at RT for 3 min, tubes were centrifuged for 20 min at 4 °C at full speed (13,800 rpm; Eppendorf Centrifuge 5417R; Eppendorf, Germany). Once the solution was separated into three layers, the uppermost RNA-containing clear layer was removed and placed in a fresh tube, to which 1:1 (v/v) isopropanol (Bio-Lab Ltd., Israel) was added to precipitate the RNA. After briefly shaking the tubes, they were incubated at RT for 5 min, after which they were centrifuged for 15 min at 4 °C at full speed (13,800 rpm). Then, the isopropanol was removed and the pellet was washed twice with 1 mL of 75% ethanol (Bio-Lab Ltd., Israel) mixed with DEPC-treated water (Biological Industries, Israel) and centrifugation for 5 min at 4 °C at full speed. After removal of ethanol, the tubes were left to dry for 10–20 min. Once dry, 20 µL of DEPC-treated water was added to each tube. Final RNA concentrations were measured using the Thermo Scientific NanoDrop 2000 spectrophotometer (Thermo Fisher Scientific, USA).

#### Reverse Transcription

Extracted total RNA was used as input for mRNA complementary deoxyribonucleic acid (cDNA) synthesis. Reverse transcription of mRNAs was conducted using the High-Capacity cDNA Reverse Transcription Kit (Thermo Fisher Scientific, USA) with random primers. The SimpliAmp thermal cycler (Applied Biosystems by Thermo Fisher Scientific, USA) was used under the following conditions: 10 min at 25 °C, 120 min at 37 °C, 5 min at 85 °C, and a final step of 4 °C until the end.

#### Real-Time Quantitative Polymerase Chain Reaction (qPCR)

mRNA expression levels were assessed using the PerfeCTa SYBR Green FastMix (Quantabio, USA), according to the manufacturer’s instructions, using the StepOnePlus Real-Time PCR System (Applied Biosystems by Thermo Fisher Scientific, USA). Thermal cycler conditions were as follows: 20 s at 95 °C, 40 amplification cycles (3 s at 95 °C to denature, and 30 s at 60 °C to anneal and extend), and a melt curve: 15 s at 95 °C to denature, 60 °C for 60 s, and an increase of 0.6 °C every 5 s (including a plate read) until reaching 95 °C. Expression values were calculated based on the comparative cycle threshold (Ct) method. mRNA expression levels were normalized to glyceraldehyde 3-phosphate dehydrogenase (Gapdh). For the detection of mRNA expression specific primers to genes of interest were used (Integrated DNA Technologies, Inc., USA) (Table 1). mRNA levels are shown as fold change (FC) relative to the control group’s expression levels. Expression profiles (measured as Ct of each sample at a constant fluorescence threshold) of the reference genes were consistent across groups, regardless of treatment.

**Table 1:** Primers used for mRNA expression analysis

Sequence 5’ to 3’	Primer direction	mRNA Target
AGGTCGGTGTGAACGGATT	Forward	*Gapdh*
TTGCCGTGAGTGGAGTCATA	Reverse	
CCATAAGCAGCCGTATGACC	Forward	*Sgk1*
ATATTGTCGTACATCTCAGCCGTG	Reverse	
CCAGAGTTGATCTTTGGAGCCACT	Forward	*Gsk3β*
CCAACTGATCCACACCACTGTCC	Reverse	
AAGGGTCTGGAGAGGACAACCTG	Forward	*Nr3c1*
GCTGGACGGAGGAGAACTCACAT	Reverse	
GGAGACCACACTGTTGCGAT	Forward	*Elmod1*
GCCAACTGTCTTCAAGCAACTG	Reverse	

#### mRNA Sequencing

 Library preparation and sequencing was performed at Macrogen Inc. (South Korea) using the TruSeq Stranded mRNA library preparation kit and the NovaSeq 6000 sequencing platform. Reads were paired-end, with a length of 100 bp per read and a throughput of 40 M reads/sample. Computational processing of the RNA-seq data was performed with the nf-core/rnaseq workflow (v3.14.0) (Ewels et al. 2020) executed under Nextflow v23.04.2 with Singularity containers to guarantee full software reproducibility. Raw FASTQ files were (i) quality-checked with FastQC v0.12.1, (ii) adapter- and quality-trimmed with Trim Galore! v0.6.7 (Cutadapt v3.4), and (iii) summarised with MultiQC v1.19. Reads were aligned to the Mus musculus reference genome (GRCm39 primary assembly, Ensembl release 111) using STAR v2.7.9a/2.7.10a with default splice-aware parameters. Alignment metrics and library complexity were assessed with Picard MarkDuplicates v3.0.0, samtools v1.17, RSeQC v5.0.2 and Qualimap v2.3. Gene-level counts were generated with featureCounts (subread v2.0.1); transcript abundances were additionally estimated with Salmon v1.10.1. Down-stream differential expression (DE) analyses were carried out in Python 3.11 with the PyDESeq2 implementation of DESeq2. Genes registering fewer than 10 total counts across the retained samples were discarded. Size factors were calculated by the median-of-ratios method, dispersion parameters were fitted and shrunk with the default empirical-Bayes procedure, and Wald tests were applied for each specified contrast. P-values were corrected for multiple testing with the Benjamini–Hochberg procedure; genes with adjusted P < 0.05 and |log2 fold-change| ≥ 2 were deemed differentially expressed. Variance-stabilised counts were obtained via PyDESeq2 and used for principal-component analysis (scikit-learn v1.3). Volcano plots and other graphics were generated with Matplotlib v3.8, Seaborn v0.13 and adjustText v0.8, with Ensembl gene IDs converted to official symbols through the MyGene.Info API.

### Statistical Analysis

Behavioral and qPCR data were statistically analyzed by the GraphPad Prism software (version 10; San Diego, CA, USA), and are presented as means ± SEM. For all calculations, Grubbs’ test was used to identify group outliers, normal distribution was tested via the Shapiro-Wilk test, and the F-test was used to compare group variances. Two-way ANOVA with Tukey’s correction for multiple comparisons, or two-way repeated measures ANOVA (when applicable), with the Fisher’s LSD test for post-hoc analyses were used for analyses. Mann-Whitney test was used as a non-parametric statistical test when needed. *p*-values ≤ 0.05 were considered statistically significant (* ≤ 0.05, ** ≤ 0.01).

## Results

### Acute, Ultra-Low Dose THC Reduces Anxiety in CMS Mice

1 month old mice underwent 5 weeks of chronic-mild stress (CMS) protocol (Willner 2017) to induce major-depression-like symptoms (CMS group), while a similar group did not undergo the CMS protocol and served as controls. On the beginning of the third week of the CMS protocol, mice received an acute, ultra-low dose THC or vehicle injection and were taken for behavioral assessment 3 weeks post treatment, once THC has cleared their systems.

**Fig. 2:**
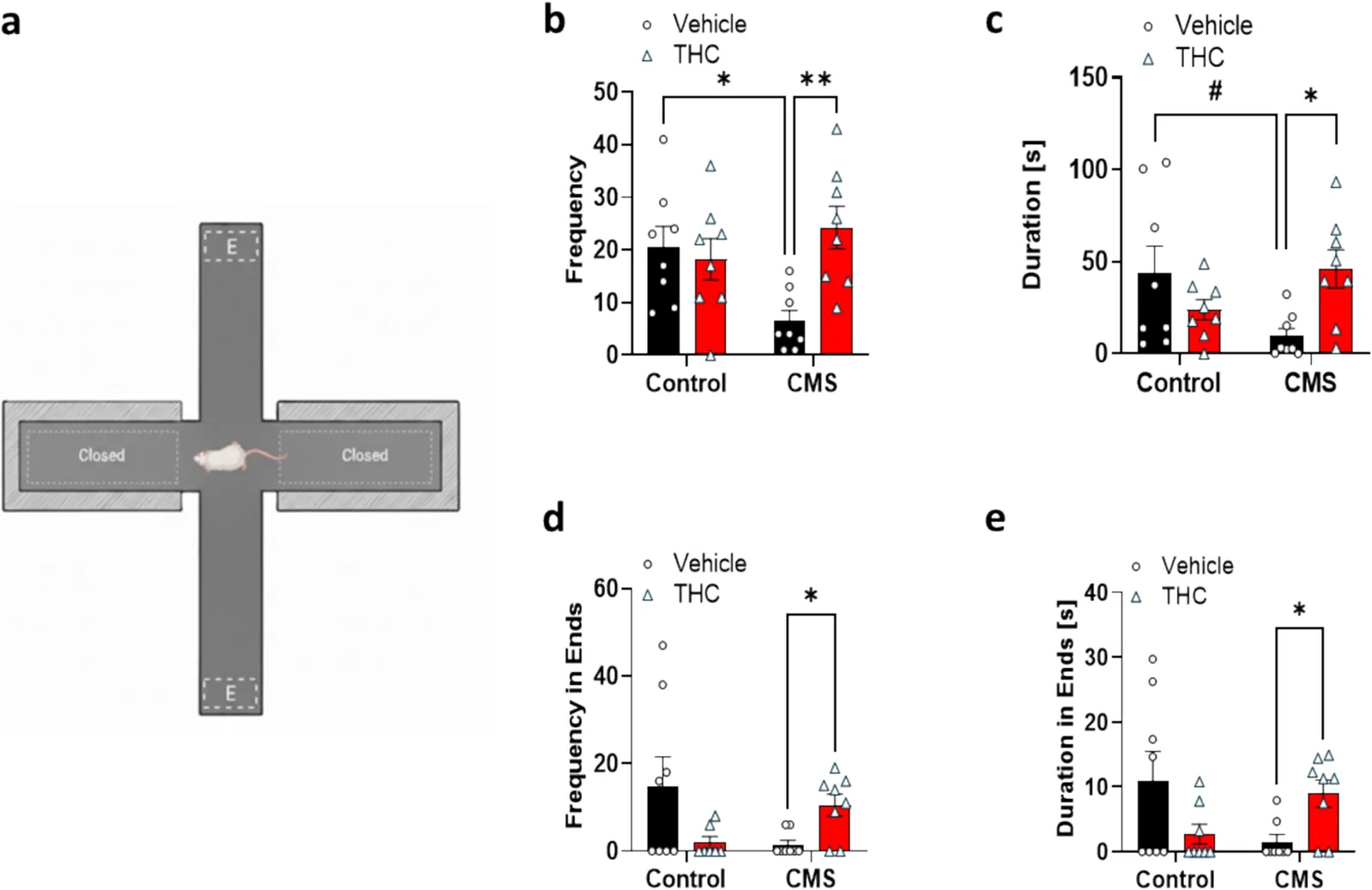
Acute, ultra-low dose THC treatment reduces anxiety levels in CMS mice, as seen in the elevated plus maze assay. (**a**) Illustration of the elevated plus maze apparatus. Decreased anxiety is measured as increased frequency or duration spent in open arms. E denotes the ends of open arms. b-e, CMS protocol increased anxiety levels in vehicle-treated mice, while THC treatment reversed this effect, as measured in frequency (**b**) and cumulative duration (**c**) spent in the open arms of the elevated plus maze, especially in the ends of the open arms **(d**,** e)**. Data are shown as means ± SEM. * *p* ≤ 0.05, ** *p* ≤ 0.01, # *p* ≤ 0.1. (**b**, **c**) two-way ANOVA with Tukey’s multiple comparisons post-hoc test; (**d**, **e**) Mann-Whitney test with Holm-Sidak correction for multiple comparisons. (**b**, **c**, **e**) *n* = 8 per group; (**d**) *n* = 7 *Control*-THC, *n* = 8 per group in rest

To assess the mice’s anxiety levels, the elevated plus maze test was utilized. Two-way ANOVA showed a significant effect for treatment (*F*(1,28) = 4.63, *p* = 0.0402) and group x treatment interaction (*F*(1,28) = 7.933, *p* = 0.008) on frequency of exploration of the open arms of the maze (Fig. 2b). *Post-hoc* analysis showed that CMS mice who received the vehicle treatment entered the open arms of the maze at a significantly lower frequency, compared to both control and the CMS mice treated with THC (*p* = 0.04, *p* = 0.007, respectively). Specifically, THC acted as an anxiolytic and normalized time spent in outer arms among the CMS mice treated with THC.

**Fig. 3:**
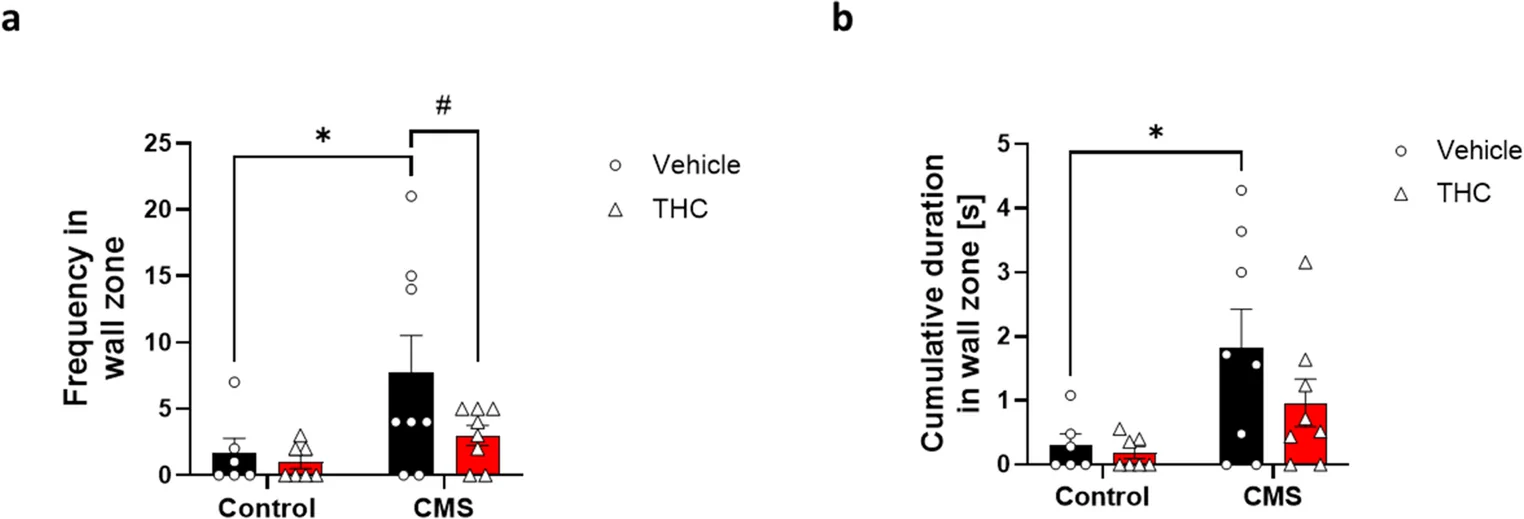
Acute, ultra-low dose THC treatment reduced anxious behavior among treated CMS-mice in the open field test. Frequency (**a**) and cumulative duration (**b**) in outer circumference of the open field test was increased in vehicle-treated CMS mice, but not in THC-treated CMS mice. Data are shown as means ± SEM. * *p* ≤ 0.05; # *p* ≤ 0.1. Two-way ANOVA with Tukey’s multiple comparisons post-hoc test. (a, b) *n* = 6 Control-Vehicle, *n* = 7 Control-THC, *n* = 8 cm. CMS = chronic mild stress

To explore the mice’s behavior in a neutral environment, we used the open field paradigm. A measure for anxiety in the open field assay is time spent in outer circumference of the arena, compared to the center of the arena. Two-way ANOVA showed a significant effect for the group measure (*F*(1,25) = 5.738, *p* = 0.024) (Fig. 3a). *Post-hoc* analysis showed vehicle-treated CMS mice spent increased time in the outer circumference of the arena (*p* = 0.038), exhibiting increased anxious behavior compared to control mice. However, THC-treated CMS mice showed a trend toward frequenting the outer circumference less compared to vehicle-treated CMS mice (*p* = 0.08), meaning they spent more time in center of arena, thus suggesting a relative reduction in anxious behavior.

### Acute, Ultra-Low Dose THC Altered Expression of Developmental- and Differentiation-Related mRNAs

Control and CMS mice were treated with either vehicle solution or acute, ultra-low dose THC, and their brains were collected for analysis of mRNA expression levels. To understand the expression patterns of genes affected by stress and treatment, total RNA was extracted from the mice’s frontal cortices and hippocampi, two brain areas that were previously implicated in CMS (Musaelyan et al. 2020).

**Fig. 4:**
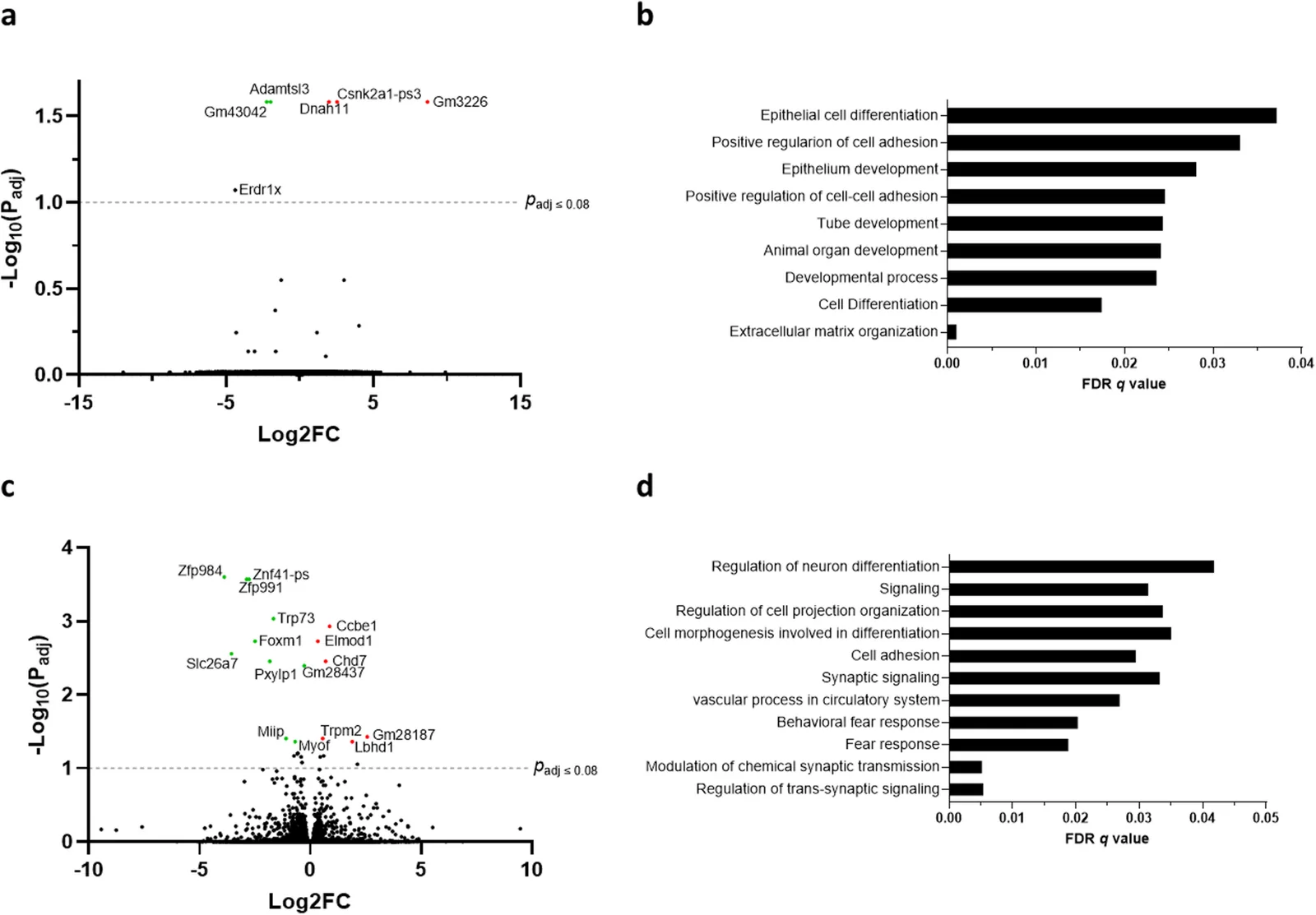
Acute, ultra-low dose THC treatment affects mRNAs involved in developmental pathways and neuronal differentiation pathways in the hippocampi of healthy young mice (controls) and CMS mice, respectively. (**a**) Volcano plot showing –log_10_P value as a function of log2 of the fold changes of mRNAs sequenced from hippocampi of healthy young mice (controls) that received acute, ultra-low THC treatment compared to vehicle-treated controls. Each dot represents mRNA of interest. Green and red indicates significantly decreased and increased expression levels respectively. (**b**) Gene ontology processes enriched among the differentially expressed mRNAs in hippocampi of THC-treated control mice compared to vehicle treated control mice. (**c**) Volcano plot showing –log_10_P value as a function of log2 of the fold changes of mRNAs sequenced from hippocampi of CMS mice that received acute, ultra-low THC treatment compared to vehicle-treated CMS mice. (**d**) Gene ontology processes enriched among the differentially expressed mRNAs in hippocampi of THC-treated CMS mice compared to vehicle treated CMS mice. *n* = 3 per group. CMS = chronic mild stress

To determine which mRNAs have been changed in the hippocampi of THC-treated mice compared to controls, we utilized a next generation sequencing technique, namely mRNA sequencing. When comparing THC- to vehicle-treated healthy young (control) mice, we saw alterations in expression levels of mRNAs related to developmental and differentiation processes (Fig. 4a, b; Table 2). Of note is *Dynein axonemal heavy chain 11* (*Dnah11*) (*p*_*adj*_=0.026) which was found to impair memory in mice through disruption of synaptic plasticity (Fu et al. 2025). Moreover, comparison between THC- and vehicle-treated CMS mice showed significant alterations in expression levels of mRNAs related to synaptic signaling, neuronal differentiation and behavioral fear response (Fig. 4c, d; Table 3).

**Table 2:** Top differentially expressed mRNAs in acute, ultra-low dose THC-treated control mice compared to vehicle-treated control mice. *n* = 3 per group

Gene symbol	Gene name	log_2_ fold change	*P*_adjusted_	Implications
*Adamtsl3*	*ADAMTS-like 3*	-2.2411	0.026188	Tumor suppressor (Nord et al. 2009)
*Dnah11*	*Dynein axonemal heavy chain 11*	1.9960	0.026188	Synaptic plasticity (Fu et al. 2025)
*Csnk2a1-ps3*	*casein kinase 2*,* alpha 1 polypeptide*,* pseudogene 3*	2.5405	0.026188	Neurodegeneration and neuroinflammation (Chen et al. 2025)

**Table 3:** Top differentially expressed mRNAs in acute, ultra-low dose THC-treated CMS mice compared to vehicle-treated CMS mice. *n* = 3 per group

Gene symbol	Gene name	log_2_ Fold change	*P*_adjusted_	Implications
*Zfp984*	*Zinc finger protein 984*	-3.88747	0.000249	Poorly characterized
*Zfp991*	*Zinc finger protein 991*	-2.87127	0.000268	Poorly characterized
*Trp73*	*Transformation related protein 73*	-1.66628	0.000925	Pro-apoptotic (Bui et al. 2009)
*Ccbe1*	*Collagen and calcium binding EGF domains 1*	0.867939	0.001175	Promotes angiogenesis (Wang et al. 2024)
*Elmod1*	*Elmo domain containing 1*	0.335891	0.001868	Cytoskeleton maintenance (Johnson et al. 2012)
*Foxm1*	*Forkhead box M1*	-2.49142	0.001868	Mitosis (Schuüller et al. 2007), neuronal fate (Pelzer et al. 2021)
*Slc26a7*	*Solute carrier family 26 member 7*	-3.55854	0.002757	ALS biomarker (Homann et al. 2025)
*Pxylp1*	*2-Phosphoxylose phosphatase 1*	-1.83281	0.003502	Proteoglycan maturation (Koike et al. 2014)
*Chd7*	*Chromodomain helicase DNA binding protein 7*	0.696575	0.003502	Chromatin remodeling (Marie et al. 2018)
*Miip*	*Migration and invasion inhibitory protein*	-1.09718	0.039251	Cytoskeleton maintenance (Wang et al. 2011)
*Trpm2*	*Transient receptor potential cation channel*,* subfamily M*,* member 2*	0.558977	0.039251	Inflammation (Chen et al. 2023)
*Lbhd1*	*LBH domain containing 1*	1.894369	0.043567	Poorly characterized
*Myof*	*Myoferlin*	-0.68598	0.043567	Cancer progression (Zhou et al. 2016)

**Fig. 5:**
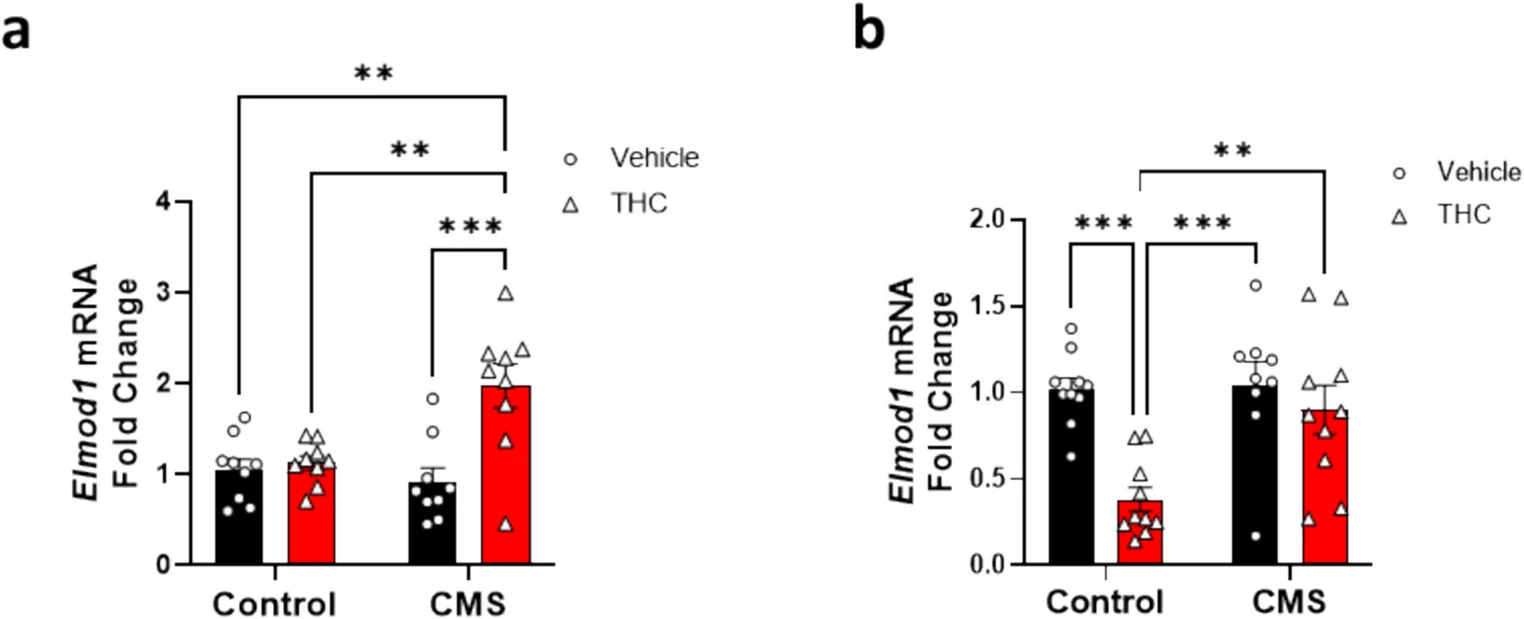
Alterations in *Elmod1* mRNA expression levels in frontal cortices and hippocampi of THC-treated CMS and WT mice. (**a**) An increase in *Elmod1* expression levels was observed in the frontal cortices of THC-treated CMS mice compared to vehicle-treated CMS mice and WT mice. (**b**) A decrease in *Elmod1* mRNA expression levels was observed in the hippocampi of THC-treated WT mice compared to vehicle-treated WT mice and CMS mice. Data are shown as means ± SEM. ** *p* ≤ 0.01; *** *p* ≤ 0.001. (a, b) Two-way ANOVA with Tukey’s post hoc test. (a) *n* = 9 per group; (b) *n* = 10 Control, CMS-THC, *n* = 9 cm-vehicle. CMS = chronic mild stress

It was previously shown that in MDD models there are neuronal cytoskeletal abnormalities that cause dendritic regression, thus harming neuronal health and decreasing synaptic connectivity (Wong et al. 2013). Our mRNA sequencing showed that the cytoskeleton stability-related *Elmo domain containing 1* (*Elmod1*) was upregulated in the hippocampi of THC-treated mice compared to vehicle-treated CMS mice, 3 weeks post treatment (*p*_*ad*j_=0.001). To understand whether there is a time- and area-selective effect on *Elmod1*, we looked into its expression levels in the hippocampi and frontal cortices of the mice via real-time qPCR, 5 weeks post treatment. Interestingly, two-way ANOVA analysis of *Elmod1* hippocampal levels showed significant effects of treatment (*F*(1,35) = 13.75, *p* = 0.0007), group (*F*(1,35) = 6.807, *p* = 0.013) and treatment x group interaction (*F*(1,35) = 5.459, *p* = 0.025) (Fig. 5b). Specifically, while *Elmod1* hippocampal levels were normalized in the CMS mice, its expression levels were significantly decreased in the THC-treated control mice hippocampi, compared to vehicle-treated control mice and vehicle- and THC-treated CMS mice (*p* < 0.001). Moreover, two-way ANOVA analysis of *Elmod1* frontal cortex levels showed significant effects of treatment (*F*(1,32) = 12.59, *p* = 0.001), group (*F*(1,32) = 5.071, *p* = 0.031) and treatment x group interaction (*F*(1,32) = 9.52, *p* = 0.004) (Fig. 5a). *Elmod1* expression levels in the THC-treated CMS mice frontal cortices was significantly increased compared to vehicle-treated CMS mice (*p* < 0.001) and control mice (*p* = 0.001).

### Acute, Ultra-Low Dose THC Altered Hippocampal Expression of Glucocorticoids-Related mRNAs

**Fig. 6:**
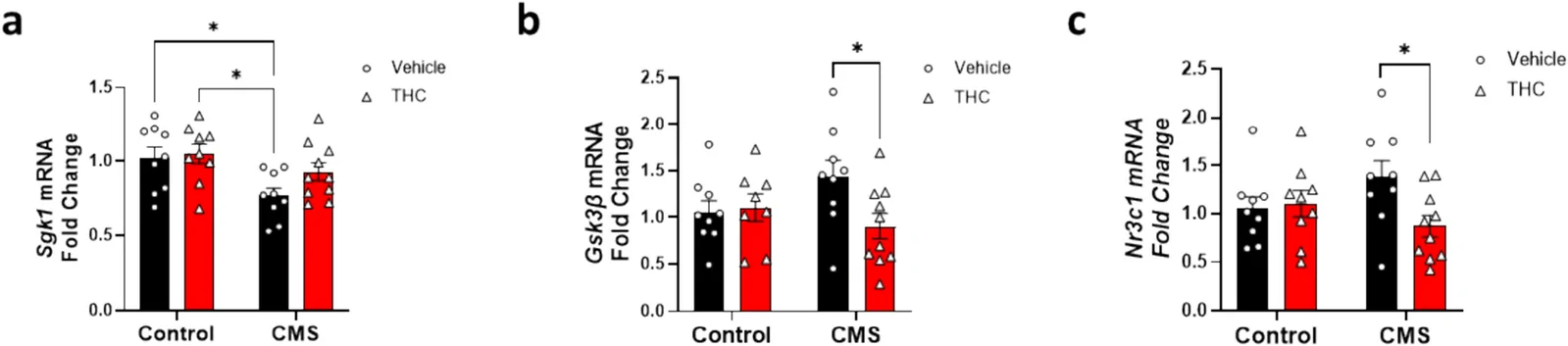
Alterations in hippocampal mRNA levels of the glucocorticoids-related gene after acute, ultra-low dose THC treatment in CMS mice. (**a**) A decrease in Sgk1 expression levels in the vehicle-treated CMS mice, but not in THC-treated CMS mice, compared to control mice. **B**,** C** mRNA expression levels of Gsk3β (**b**) and Nr3c1 (**c**) were significantly decreased in the hippocampi of THC-treated CMS mice compared to vehicle-treated CMS mice. Data are shown as means ± SEM. * *p* ≤ 0.05. (a-c) Two-way ANOVA with Tukey’s post hoc test. (a) *n* = 9 control, CMS-vehicle, *n* = 10 cm-THC; (b) *n* = 10 control, CMS-THC, *n* = 9 cm-vehicle; (c) *n* = 9 Control-vehicle, CMS-vehicle, *n* = 8 Control-THC, *n* = 10 cm-THC. CMS = chronic mild stress

Stress-induced glucocorticoids increase was previously shown to promote dendritic regression, followed by the decrease in spine density and synaptic connectivity (Chen et al. 2008; Magarin˜os and McEwen 1995). Moreover, previous research has linked acute, ultra-low dose THC treatment to changes in glucocorticoid-related genes, specifically *Serum and glucocorticoid kinase 1* (*Sgk1*) (Nitzan et al. 2026; Shapira et al. 2023). Real-time qPCR analysis revealed hippocampal alterations in several glucocorticoid-related mRNAs; Firstly, two-way ANOVA of *Serum and glucocorticoid kinase 1* (*Sgk1*) hippocampal levels showed a significant group effect (*F*(1, 33) = 8.892, *p* = 0.005), with *Sgk1* significantly reduced in the hippocampi of vehicle-treated CMS mice (*p* = 0.037), a reduction that was prevented in THC-treated CMS mice (Fig. 6a). *Sgk1* is transcriptionally regulated in neurons in response to stress and is part of the negative-feedback loop of glucocorticoids signaling, thus making it a critical player in the HPA-axis pathway (Doyle et al. 2020). Another regulator of glucocorticoid response, *Glycogen synthase kinase-3β* (*Gsk3β*) (Rubio-Patiño et al. 2012), showed a group x treatment interaction marginal effect (*F*(1,32) = 3.775, *p* = 0.06), with *Gsk3β* levels being increased in vehicle-treated CMS mice compared to THC-treated CMS mice (*p* = 0.027) (Fig. 6b). Lastly, two-way ANOVA showed a group x treatment interaction effect (*F*(1,33) = 4.21, *p* = 0.048) in the expression levels of the GC-R-encoding gene, *Nuclear receptor subfamily 3 group c member 1* (*Nr3c1*) in the HIP. *Post-hoc* analysis showed *Nr3c1* to be transcriptionally increased in vehicle-treated, but not CMS-treated mice hippocampi (*p* = 0.055) (Fig. 6c). When using another, less comprehensive statistical measure, such as multiple comparisons between groups, where we compare treatment directly within the control/CMS groups and not between them, we receive a p-value of 0.012. These results show that acute, ultra-low dose THC attenuated glucocorticoids-mediated transcription in the hippocampi of CMS mice.

## Discussion

7 weeks-old control and CMS mice received acute, ultra-low dose THC treatment, and were taken for behavioral assessment of anxiety. THC-treated CMS mice showed reduced anxious behavior in the elevated plus maze and open field tests, compared to vehicle-treated CMS mice, showing the anxiolytic potential of acute, ultra-low dose THC. While previous research has showed that THC can possess anxiolytic properties through chronic treatment with low doses (Andrade et al. 2019; Bluett et al. 2017; Harte-Hargrove and Dow-Edwards 2012; Schreiber et al. 2019; Uttl et al. 2018), the current results show that a single treatment of ultra-low dose THC can be sufficient to induce anxiolytic effects.

mRNA sequencing of hippocampal samples of THC- and vehicle-treated CMS mice 3 weeks post treatment showed alterations in expression levels of mRNAs involved in synaptic signaling, neuronal differentiation and behavioral fear response, while comparison between THC- and vehicle-treated control mice showed alterations in mRNAs involved in developmental and differentiation processes. Among the CMS group, one of the differentially expressed mRNAs was *Elmod1*, which is a GTPase-activating protein that belongs to the ARF family of small G proteins (Johnson et al. 2012). It was found to be expressed in the cerebellum, HIP, hypothalamus, prefrontal cortex, striatum and retinal ganglion cells (Johnson et al. 2012; Pandey et al. 2014). Mutations in *Elmod1* were linked to impairments in actin cytoskeleton stability and maturation, resulting in deafness and balance defects (Johnson et al. 2012). Specifically, *Elmod1* regulates Arf6 through GAP activity (Krey et al. 2018). Inactivation of ARF6 in the apical surface of hair cells is critical for the maintenance of the cuticular plate and stereocilia structures (Krey et al. 2018). Arf6 acts by regulating actin rearrangements, and it was found that decreasing Arf6 activation increases anterograde axonal transport (Eva et al. 2012) and enhances axonal elongation and branching in developing hippocampal neurons (Hernández-Deviez et al. 2004). Interestingly, post-translational modifications of actin and microtubule filaments were observed in rodent models of depression (Wong et al. 2013), as well as changes in levels of proteins related to cytoskeleton properties (Coumans et al. 2016). In the current study we observed an increase in frontal cortices *Elmod1* expression levels in CMS mice, which was associated with reduced anxious behavior. Thus, *Elmod1* overexpression may hold anxiolytic potential, perhaps by the inactivation of Arf6 activity. Further research should be made into the effect of acute, ultra-low dose THC on Arf6 activity in the brain.

Additionally, whole exome sequencing in a patient with intellectual disability showed the existence of a mutation in *Elmod1*, linking it to cognitive functions (Miryounesi et al. 2019). Our results showed that acute, ultra-low dose THC treatment was associated with reduced hippocampal *Elmod1* expression levels in control mice. This is noteworthy because previous research has shown acute, ultra-low dose THC to induce cognitive impairments in young, healthy mice (Tselnicker et al. 2007; Senn et al. 2008; Amal et al. 2010). Therefore, it is possible that *Elmod1* downregulation can be linked to cognitive impairments. Further studies should focus on the involvement of *Elmod1* in cognitive functions, MDD and other stress-related disorders.

*Sgk1* is a serine/threonine kinase which is transcriptionally regulated in neurons by glucocorticoids in response to stress (Doyle et al. 2020). Curiously, while we saw a decrease in hippocampal *Sgk1* expression levels in the vehicle-treated CMS group, many studies link an increase in *Sgk1* levels to depressive symptoms in both murine models and humans (Anacker et al. 2013; Dattilo et al. 2020; Millette et al. 2025; Zhang et al. 2016). Nevertheless, Licznerski et al. show that *Sgk1* expression is reduced in the postmortem PFC of post-traumatic stress disorder (PTSD) subjects, and that *Sgk1* inhibition in the PFC of rats resulted in anxious behavior (Licznerski et al. 2015). Furthermore, inhibition of *Sgk1* was shown to enhance the production of pro-inflammatory cytokines, and aggravate the inflammatory response in microglia, showing an anti-inflammatory potential for *Sgk1* (Inoue et al. 2016; Zhou et al. 2015). Thus, it is possible that the immediate effect of CMS is downregulation of *Sgk1* and the consequent inflammatory response. Interestingly, this downregulation was not observed in THC-treated CMS mice, suggesting the importance of balanced *Sgk1* levels and that acute, ultra-low dose THC protected against this decrease and inflammatory response, thus providing the observed anxiolytic effects.

*Gsk3β* is also a serine/threonine kinase and a master regulator of multiple signaling pathways, such as transcription, mitochondrial function, and cytoskeleton maintenance, making it critical for sustainment of cell homeostasis (Duda et al. 2020). It is a key regulator of neuronal plasticity, neurogenesis, and stress-responsive signaling pathways in the brain, processes that are frequently disrupted in MDD (Aceto et al. 2020; Boku et al. 2018; Oh et al. 2010). Postmortem analysis of human hippocampal tissue revealed elevated transcriptional abundance (increased *GSK3β* mRNA expression) in patients with MDD, supporting a role for this kinase in depression pathophysiology (Oh et al. 2010). Previous research demonstrated that *Gsk3β* is increased at both the expression level and its enzymatic activity in CMS mice brains, and that its pharmacological inhibition was correlated with the reduction in inflammatory processes and depressive-like behaviors (Ebeid et al. 2021; Peng et al. 2018). In a rat model of maternal deprivation, depressive symptoms were linked to cytoskeletal alterations through post-translational regulation of the AKT/GSK3β/CRMP2 signaling cascade (Wei et al. 2021). Together, these findings suggest that both elevated expression and functional hyperactivation of Gsk3β may promote depressive phenotypes. Our results showed that acute, ultra-low dose THC treatment reduced *Gsk3β* mRNA expression levels in the hippocampi of CMS mice compared to vehicle-treated CMS mice, indicating a downregulation at the transcriptional level. Previous research showed low-dose THC to have beneficial effects on behavior in a phencyclidine (PCP) rat model of the negative symptoms of schizophrenia, an effect that was associated with post-translational modulation, namely a decrease in phosphorylated Gsk3β levels in the rats’ nucleus accumbens, without alterin gtotal Gsk3β protein levels (Seillier et al. 2020). Together with our results, low-dose THC treatment can be linked to *Gsk3β*. However, our results are limited to the transcriptional level, showing a decrease in total *Gsk3β* expression levels in the hippocampi of treated CMS mice, rather than direct post-translational changes in Gsk3β phosphorylation status or functional kinase activity.

Notably, *Gsk3β* is directly inhibited by *Sgk1* (Wyatt et al. 2006). A study in a rat model for PTSD showed that single prolonged stress upregulates AKT/Gsk3β and downregulates Sgk1/ERK1/2, further linking *Gsk3β* and *Sgk1* to stress-induced pathophysiology (Wei et al. 2019). Previous research showed that acute, ultra-low dose THC treatment exerts its neuroprotective effect by alterations of ERK2 activation in multiple brain areas, including the HIP (Fishbein et al. 2012). ERK is known to have an important role in cell survival and death, and synaptic plasticity (Iroegbu et al. 2021), thus it is possible that acute, ultra-low dose THC exerts its anxiolytic effects through modulation of ERK, and future studies should unfold its involvement.

We also show that acute, ultra-low dose THC prevented the increase in *Nr3c1* expression levels in the hippocampi of CMS mice, 3 weeks post treatment, at the cessation of the CMS manipulation. *Nr3c1* encodes the GC-R and has a regulatory role in HPA-axis function (Park et al. 2019). Accordingly, Wei et al. show that treatment with the flavonoid icariin ameliorated the increase in *Nr3c1* expression in the PFC of CMS rats, along with reduction of depression-like behaviors (Wei et al. 2016). Another study shows that early life stress caused increases in *Nr3c1* expression levels in the paraventricular nucleus, and hypermethylation at CpG-dense islands at the promoter of *Nr3c1* (Bockmühl et al. 2015). Thus, stress is linked with increases in *Nr3c1* expression levels, which can be ameliorated by acute, ultra-low dose THC treatment. Current evidence does not support direct molecular interaction between THC and *Nr3c1*. Instead, THC may modulate glucocorticoid receptor function indirectly through CB1-R-mediated regulation of HPA axis activity, thereby altering glucocorticoid availability and downstream GC-R signaling in stress-responsive brain regions such as the HIP (Barna et al. 2004; Cota 2008; Shoshan and Akirav 2017) Moreover, given that *Sgk1* has been shown to enhance GC-R activation and signaling (Anacker et al. 2013), the normalization of *Sgk1* expression despite reduced Nr3c1 levels may indicate a restoration of glucocorticoid signaling efficiency rather than a simple dependence on receptor abundance.

**Fig. 7:**
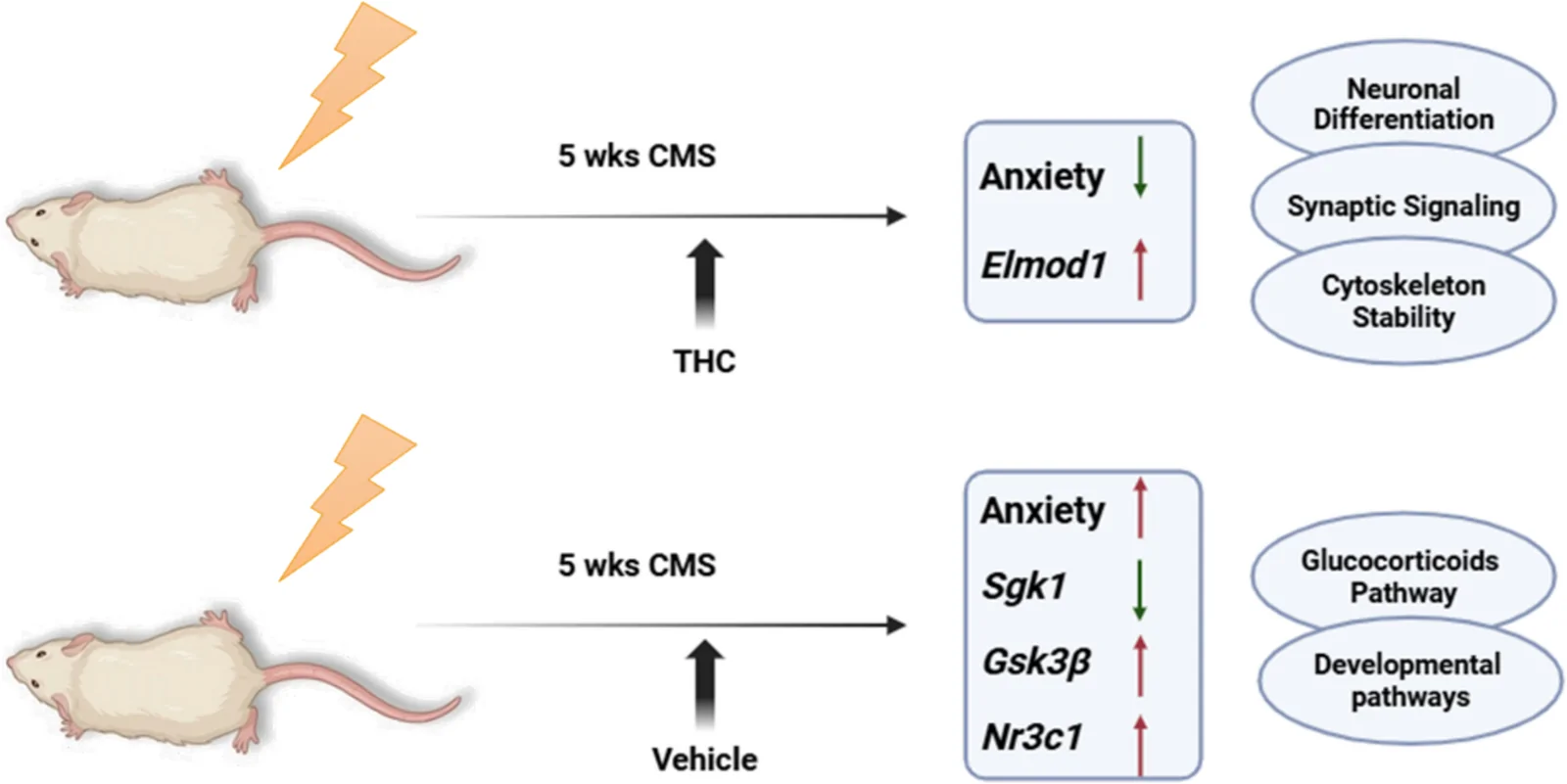
Alterations in mRNA levels of multiple pathways in CMS mice, treated with ultra-low dose THC or vehicle. one-month old mice underwent chronic mild stress (CMS) for 5 weeks, during which they were treated with ultra-low dose THC or vehicle. Transcriptomic analysis of the mice’s hippocampi and frontal cortices showed that in the ultra-low dose THC-treated mice there were changes in transcripts related to neuronal differentiation, synaptic signaling and cytoskeleton stability, while in the vehicle-treated group there were changes in transcripts related to the glucocorticoids pathway and development

To summarize, acute, ultra-low dose THC was able to reduce anxious behavior in CMS mice, as shown in the behavioral tests for anxiety. This effect was shown to be associated with changes in the expression levels of *Gsk3β*, *Nr3c1* and *Elmod1*, thus associating THC with the glucocorticoid pathway and cytoskeleton stability (Fig. 7). Moreover, as the mRNA sequencing results show, there are additional neuronal-differentiation and synaptic signaling-related mRNAs involved in the anxiolytic effect exerted by acute, ultra-low dose THC treatment. Lastly, previous research has shown that acute, ultra-low dose THC treatment can cause cognitive impairments in healthy, young mice (Senn et al. 2008; Tselnicker et al. 2007). Our results show that the same treatment caused alterations in neurodevelopment-related mRNAs, including the hippocampal downregulation of *Elmod1*. Further experiments should be performed to better assess the molecular pathways underling this context-dependent effect of acute, ultra-low dose THC.

Nevertheless, this study is not without its limitations. Our data reflect changes in mRNA expression levels that may not be seen at the protein level and do not address protein modifications that might occur. Additionally, the mRNAs we screened for are taken from bulk tissues and are not cell-specific. Only the frontal cortex and HIP were screened, and not other brain areas that may be relevant to the effects of CMS and THC on gene expression, such as the amygdala. Another limitation of this study is the small RNA-seq sample size, which reduces power to detect moderate transcriptional changes and may also make differential-expression results more sensitive to outliers or residual technical variation. Therefore, these findings should be interpreted as exploratory and require validation in larger independent cohorts. Further experiments should be performed to better assess the molecular pathways underling the effects of acute, ultra-low dose THC in the MDD mouse model. Moreover, additional behavioral tests should be performed to expand the behavioral characteristics of acute, ultra-low dose THC treatment on different aspects of anxious and depressive-like behaviors in mice of various strains, keeping in mind the inherent difference between humans and mice, and the translational hurdle that needs to be addressed.

The age of the mice used should also be pointed out; CMS protocol started at adolescence and ended at young adulthood, with THC being injected at mid-adolescence, and the behavior and molecular changes tested at adulthood. Kasten et al. previously showed that 10 mg/kg of THC had anxiogenic effects in adult mice, and a milder anxiogenic affect in adolescent mice (Kasten et al. 2017, 2019). However, these studies used a significantly higher dose and dosage of THC and a different mouse strain (C57BL/6J and DBA/2J).

While further studies should be made to deeply understand the molecular mechanism underlying the effects of acute, ultra-low dose THC, with emphasis on the glucocorticoid pathway and cytoskeleton stability, our results show the potential of acute, ultra-low dose THC treatment in ameliorating anxious behavior through distinct mechanisms at the mRNA level. The use of an acute, ultra-low dose holds substantial translational value for clinical adoption, as compliance may be higher when treatment is given only once. Ultimately, this work shows that an extremely low dose of THC is sufficient to produce behavioral effects without requiring the chronic, high-dose regimens typically needed to achieve similar outcomes.

## Data Availability

No datasets were generated or analysed during the current study.
